# Assessing the Feasibility of an Open-Source Virtual Reality Mirror Visual Feedback Module for Complex Regional Pain Syndrome: Pilot Usability Study

**DOI:** 10.2196/16536

**Published:** 2021-05-26

**Authors:** Andrea Stevenson Won, Ariana C Barreau, Mark Gaertner, Tristan Stone, Joshua Zhu, Cheng Yao Wang, Sean Mackey

**Affiliations:** 1 Department of Communication Cornell University Ithaca, NY United States; 2 Carle Illinois College of Medicine University of Illinois Urbana-Champaign Urbana, IL United States; 3 Department of Neurology University of California, Los Angeles Los Angeles, CA United States; 4 Department of Computer Science Cornell University Ithaca, NY United States; 5 Department of Information Science Cornell University Ithaca, NY United States; 6 Stanford Neuroscience and Pain Lab Stanford University Palo Alto, CA United States

**Keywords:** virtual reality, pain, complex regional pain syndrome, CRPS, open source, mirror visual feedback

## Abstract

**Background:**

Complex regional pain syndrome (CRPS) is a rare and severe chronic pain condition, with effective treatment options not established for many patients. The underlying pathophysiology remains unclear, but there is a growing appreciation for the role of central mechanisms which have formed the basis for brain-based therapies such as transcranial magnetic stimulation and mirror visual feedback (MVF). MVF has been deployed in the treatment of CRPS using both conventional mirrors and virtual reality (VR).

**Objective:**

The aim of this study was to further investigate the use of VR in the treatment of patients with unilateral upper limb CRPS. VR has the potential advantage of more flexible and more motivating tasks, as well as the option of tracking patient improvement through the use of movement data.

**Methods:**

We describe the development, acceptability, feasibility, and usability of an open-source VR program MVF module designed to be used with consumer VR systems for the treatment of CRPS. The development team was an interdisciplinary group of physical therapists, pain researchers, and VR researchers. Patients recruited from a pain clinic completed 3-5 visits each to trial the system and assessed their experiences in pre- and post-treatment questionnaires.

**Results:**

All 9 (100%) participants were able to use the system for 3, 4, or 5 trials each. None of the participants quit any trial due to cybersickness. All 9 (100%) participants reported interest in using the module in the future. Participants’ reported average pain scores in the affected limb were not significantly different from baseline during treatment or after treatment (*P*=.16). We did not find a statistically significant effect on participants’ self-reported average pain scores.

**Conclusions:**

We propose that this module could be a useful starting point for modification and testing for other researchers. We share modifications to make this module usable with standalone headsets and finger tracking. Next steps include adapting this module for at-home use, or for use with participants with lower limb pain.

## Introduction

Complex regional pain syndrome (CRPS) is a rare and severe chronic pain condition, defined as “continuing pain...disproportionate to any inciting event,” accompanied by an array of varying signs and symptoms (sensory, vasomotor, sudomotor/edema, or motor/trophic features) which do not fit other diagnoses [1-3]. A Cochrane Review found low level of quality of evidence for CRPS treatments [4], with current treatment guidelines based largely on expert experience, case reports, open-label trials, and pilot studies [3]. Thus, many patients are without established, effective treatment options [5]. While the underlying pathophysiology remains unclear, there is a growing appreciation for the role of central mechanisms which have formed the basis for brain-based therapies such as transcranial magnetic stimulation [6] and mirror visual feedback (MVF). MVF [7] has been deployed in the treatment of CRPS using both conventional mirrors [8] and virtual reality (VR) [9-11]. VR has the advantage of more flexible and more motivating tasks [10-12], the ability to be used at home [13], and the option of tracking patient improvement through the use of movement data [11,12,14]. However, barriers of cost and difficulty of adoption have prevented this technology from being widely used.

Given the advent of inexpensive, portable consumer VR systems, systematically testing the potential of these systems for the treatment of CRPS and other disorders has become a possibility for many researchers who have not previously used VR in medicine. Virtual environments for therapeutic purposes can be created using game engines such as Unity 3D or Unreal. These virtual environments can be modified to allow researchers to investigate the effects of avatar realism, limb swapping, changing the appearance of the targets, changing the environment around the targets, etc. However, because many researchers working with clinical populations have limited experience building virtual environments, there is a significant barrier to entry.

To provide a tool for the research community as well as for our own research, we developed an open-source VR mirror module that could be used for our own experiments. We deployed this module in a pilot study for patients with chronic unilateral upper limb CRPS.

This pilot study was a feasibility study to test the usability of the VR system and its acceptance, ease of use, and patient willingness to engage. We evaluated these goals by examining participant retention, as well as qualitative comments on general usability and interest in engaging with the module at home. Participants’ reported average pain scores in the affected limb were not significantly different from baseline after treatment (*P*=.16). Furthermore, all participants completed the required sessions for this study and qualitative measures indicated that participants were interested in continuing the therapy at home.

## Methods

### Virtual Reality Application

**Figure 1:**
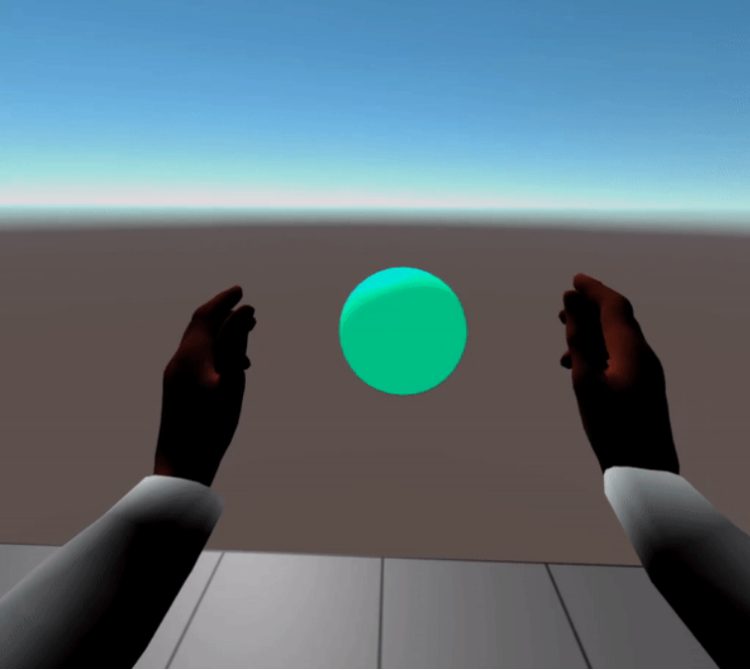
Image representing participant in VELOS MVF with a mono version of their first person perspective.

A team of pain researchers and VR researchers created a simple open-source VR application for patients with CRPS using the game engine Unity 3D. This application is compatible with a range of consumer VR systems that use head-mounted displays and hand controllers or hand tracking to allow users to interact with virtual content by controlling avatars. Figure 1 shows an example of a user’s view of their avatar’s hands which are controlled by their movements.

As in conventional MVF, movement from the patient’s uninjured hand was transformed over the midline to symmetrically animate their avatar hand on the injured side. Thus, patients’ uninjured hands could control the movements of both avatar hands, just as in conventional mirror therapy.

### Participants

In the summer of 2017, 110 adult patients in the database of a pain clinic at a large United States university with a diagnosis of unilateral CRPS in an upper extremity were contacted to ask if they would be interested in participating in a pilot study to test the feasibility of a novel VR intervention. Eligible patients had average pain level reported on a Visual Analog Scale as greater than or equal to 4 out of 10, were on a stable treatment plan for the month preceding the study, and could commit to multiple visits. A total of 9 patients (6 female and 3 male) were recruited within the 2-month time span set for the study. Among the 9 patients, 7 (78%) identified as Caucasian, 1 (11%) as Asian, and 1 as “other” (11%). Their ages ranged from 19 to 60 years, with the median being 42 and mean 44. Their education level ranged from a high-school diploma to graduate degrees, and their income ranged from under US $10,000 to US $80,000 or more. All 9 patients were right handed, with 3 having CRPS on the left upper extremity and 6 with right upper extremity CRPS. All patients signed informed consent in the clinic, received US $20 per hour of visit time, and the experiment was approved by the Stanford Institutional Review Board. The consent form, along with all other study materials, is shown in Multimedia Appendix 1.

Table 1 shows participants’ symptom onset and duration.

**Table 1:** Participants’ CRPS symptom onset and duration.

Patient ID	Symptom onset date	Duration (from the onset of CRPS^a^ to the baseline assessment date)
2	August 16, 2008	8 years and 11 months
3	May 15, 2010	7 years and 2 months
4	December 12, 2012	4 years and 7 months
7	January 11, 2015	2 years and 6 months
8	June 1, 2013	4 years and 2 months
9	September 6, 2016	10 months and 29 days
10	August 7, 2016	1 year and 16 days
11	March 28, 2013	4 years and 4 months
12	February 2, 2015	4 years and 6 months

^a^CRPS: complex regional pain syndrome.

### Materials

To make this environment as accessible to as many users as possible, it was built to accommodate both the hardware systems currently supported by the Steam VR plug-in in the Unity game engine. At the time of the experiment, these were the Oculus Rift/Touch system [14] and the HTC VIVE system [15], both of which cost less than US $400 and could be deployed using a “VR-ready” laptop as seen in Multimedia Appendix 2. The application can accommodate, but does not require, room-scale tracking. Since this initial study, the project has been revised to be used with standalone headsets that include hand and finger tracking. Original and revised projects and documentation can be found in [16].

### Baseline Survey

All visits took place in July and August of 2017. After the screening interview, participants completed a baseline survey at the clinic through the institution’s REDCap account. This survey consisted of a set of demographic and baseline measures, including the PROMIS measures of pain and daily function [17]. This survey was repeated after the last session, and 1 month after the study. All survey instruments are provided in Multimedia Appendix 1.

### Procedure

Participants then returned to the clinic weekly for a minimum of 4 sessions of immersive VR, with an optional fifth visit based on participant interest and availability. After each session, participants completed a daily pain survey (modified from the Brief Pain Inventory [18]) and a presence questionnaire derived from Witmer and Singer [19].

Participants were seated in a swivel chair in front of the computer and a researcher (AB) helped them to don the headset and hold the hand controller in their unaffected hand. Avatars were scaled to the participants’ seated height and the color of the arms and hands was selected at the beginning of each session. For participants suffering from CRPS of the right upper limb, both avatar hands were controlled using the left controller. For participants suffering from CRPS of the left upper limb, this was reversed and both avatar hands were controlled using the right controller. It was too painful for some participants to hold the tracker with their injured hand, so all participants held only 1 tracker on their uninjured side.

The researcher (AB) then began the session by pressing the spacebar key. Each time the key was pressed, one of a series of 18 targets of 3 different sizes (6 large, 6 medium, and 6 small) appeared in random order in the midline of the participant’s field of view. Participants were asked to bring both the injured and uninjured hands together to contact the target. When an avatar hand contacted the target, a chime sounded, and the target object disappeared. Participants completed as many sets of target hitting as they wished (mean 7.5 [SD 2.25]). For the first 2 participants, the protocol differed slightly for their first 2 visits, in that they were only allowed to complete 2 sets.

### Measures Taken at Each Session

#### Pain, Physical Activity, Mood, and Quality of Sleep

**Figure 2:**
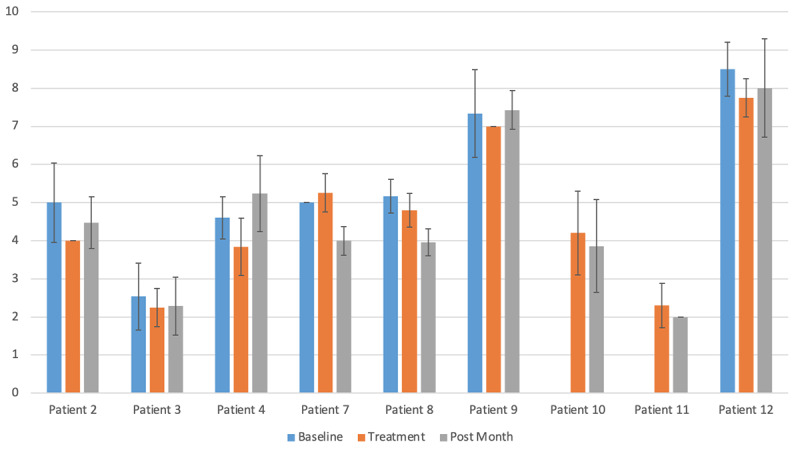
Patient ratings of average pain in affected CRPS limb. Ratings were on a 0-10 scale, with 10 being the most pain. Ratings were averaged before treatment, during treatment, and at one month post-treatment.

Before completing the target-hitting task, participants reported their average level of pain throughout their body and in their affected CRPS limb, their level of physical activity, their mood, and the quality of their sleep over the past 24 hours, as well as their CRPS-related pain at that moment and any qualitative responses (Figure 2). These data are described in Multimedia Appendix 3.

#### Movement Data

Participants’ movements were recorded at a rate of 30 times per second. As each target was hit, the time and the position of the trackers at the time of contact were also recorded.

#### Cybersickness Measures

When considering the effectiveness of such a system in a clinical setting, it is important to assess its usability. Thus, we included a measure of cybersickness, also known as simulator sickness, which occurs when exposure to a virtual environment causes symptoms similar to motion sickness.

## Results

All 9 participants completed 3, 4, or 5 sessions, and all were interested in continuing the therapy at home. Detailed results are presented in the following sections.

### Baseline Survey

The results of the baseline surveys can be found in Multimedia Appendix 3.

### Pain, Physical Activity, Mood, and Quality of Sleep

Several participants commented positively on the sessions, for example “VR helped hand pain!” and “I’m more likely to use my affected arm than I was before the study. Even my family noticed.” However, using a linear mixed model with Bonferroni-corrected α of .01 for multiple comparisons, there were no statistically significant differences over time on average or highest pain of the affected limb or body, or on physical activity, mood, or quality of sleep. Table 2 shows these results.

**Table 2:** Nonsignificant effects of time on patient self-reported outcomes using linear mixed models, predicting each outcome measure averaged at each time point and including participant ID as a random effect. Alpha adjusted to .007 using Bonferroni correction for multiple comparisons. We note that this study was not powered to detect efficacy.

Outcome measure	*P* value
How would you rate your average level of pain in your affected CRPS limb over the past 24 hours?	.13
How would you rate your average level of pain in your throughout your body over the past 24 hours?	.76
How would you rate your highest level of pain in your affected CRPS limb over the past 24 hours?	.04
How would you rate your highest level of pain throughout your body over the past 24 hours?	.64
How would you rate your level of physical activity over the past 24 hours?	.75
Rate your overall mood for the past 24 hours.	.60
How would you rate the quality of your sleep over the past 24 hours?	.84

### Movement Data

Most consumer VR systems contain trackers on the head-mounted display and 2 hand controllers, each of which captures both position (X,Y,Z) and orientation (pitch, yaw, roll) data at each frame. The default setting in our current module is to record every third frame, for a rate of approximately 30 frames per second. However, the frame rate can be modified to record at a higher or lower rate. We saved movement data for each tracker to a separate CSV file at the end of the experiment. We note that the data we recorded reflect the actual positions of the single tracker held by the participants, and the head-mounted display that they wore on their heads.

In this pilot study, participants’ head and uninjured hand movements were tracked. Participants were not asked to hold or attach a hand sensor to their injured hand, on the grounds that this might exacerbate their injury. Thus, the data collected from these sensors cannot inform us about how participants may or may not have moved their injured arm during the experiment. Data collected from the other 2 sensors can be used to examine changes in movement over the course of the following sessions, and can also be compared with other, self-reported measures. A representative example of movement data can be found in Multimedia Appendix 4.

### Cybersickness

The majority (7 participants) reported no cybersickness, with 2 participants rating their cybersickness as “slight.” No participants quit any trial due to its effects.

## Discussion

### Principal Findings

In this pilot study, we tested the feasibility, ease of use, and patient willingness to engage with an open-source MVF VR module for CRPS. All 9 participants were able to use the system for 3, 4, or 5 trials each, all participants stated that they were interested in an at-home trial if one were made available, and no participants stopped any trial due to cybersickness. We did not find a statistically significant effect of trial on participants’ self-reported average pain scores, but this is not surprising given that the study was not intended or powered to detect a clinical signal for efficacy.

### Limitations

Because of the small number of participants, we cannot draw conclusions about efficacy nor do we have enough information to discuss generalizability. In addition, participants were not blinded to treatment, and the researcher working directly with the patients (AB) was also not blind to the purpose of the study.

### Next Steps

Because in this study, participants only held the trackers in their *un*injured hand, we could not directly track improvement in movement on the injured side.

Although hand tracking was not available at the time this study was conducted, we have since updated the project to include markerless hand tracking, including finger tracking through the Oculus Quest. This can facilitate tracking of the injured hand, and may also improve the overall patient experience. Future work should examine whether markerless tracking and finger tracking improve patient outcomes.

Other potential easy modifications include changing the appearance of the avatar body, changing how the avatar limbs are controlled, and changing the appearance and placement of the targets. We have made this project open source with the goal of encouraging further exploration of deploying MVF in immersive VR using consumer systems.

This pilot study measured participant pain scores using a daily pain survey modified from the Brief Pain Inventory [18]. Future studies may use other standardized pain indicators, for example, Pain Self-Efficacy Questionnaire (PSEQ) [20], EuroQol-5D (EQ-5D) [21], or Pain Catastrophizing Scale (PCS) [22], as well as analyzing improved function as an indicator of positive outcome.

All 9 participants in our initial pilot study were interested in an at-home trial if one were made available. While at-home VR systems are still out of reach for most consumers, such systems can increase accessibility [23]. An at-home module would allow patients to use the module at will and could also be a useful channel of communication between the clinician and the patient. In such a system, safety and social interaction must be fully considered. Thus, any consent form for such an environment should reiterate potential risks and protection of patients’ data must also be assured [24].

We note that this software is not a commercial system or patient-ready application. Rather, it is designed to be used as a potential starting point for research for other clinicians interested in exploring potential therapeutic uses of immersive VR for visual feedback on movement. We look forward to the contributions of other researchers in this area.

## Appendix

Multimedia Appendix 1 All study materials, including consent form, interview protocol and survey questions.

Multimedia Appendix 2 A video of two research assistants using the system to simulate the patient experience.

Multimedia Appendix 3 Anonymized data set containing participants' self-reported pain ratings before and after the virtual reality sessions.

Multimedia Appendix 4 A representative example of participant movement data.

## Abbreviations

CRPS: complex regional pain syndrome
EQ-5D: EuroQol-5D
MVF: mirror visual feedback
PCS: Pain Catastrophizing Scale
PSEQ: Pain Self-Efficacy Questionnaire
VR: virtual reality
