# Supplementary material for: Assessing the Feasibility of an Open-Source Virtual Reality Mirror Visual Feedback Module for Complex Regional Pain Syndrome: Pilot Usability Study
Source: J Med Internet Res. 2021 May 26;23(5):e16536. doi: 10.2196/16536 (PMC8190641; doi:10.2196/16536)
Supplement: Multimedia Appendix 1 [file jmir_v23i5e16536_app1.pdf]

VR-CRPS Screening Survey

The purpose of this screening interview is to see if you meet the criteria for taking part in our research study of the chronic pain condition Complex Regional Pain Syndrome (CRPS), also known as Reflex Sympathetic Dystrophy (RSD). I'm going first go through a list of questions to see if you are eligible for this study, and then I'll tell you more about the study so you can decide whether you are interested in participating.

This interview will take approximately 10 minutes. The risk of taking part in this interview is very small. The screening interview is not designed to ask you for sensitive personal information, but it is possible that some people may feel uncomfortable answering these questions with a person they do not know. You may choose not to answer these questions. You also may choose to stop participating in this interview at any time – there is no penalty for stopping the interview. If you would like to stop this interview at any time, please let me know.

Information about you that you give me during this interview will be kept as confidential as possible as required by law. It is possible that the Food and Drug Administration, and other federal and state authorities, may inspect this record. There is a small risk that people outside our research team could learn this information, but we do our best to keep all your information confidential.

If you decide you're not interested, you can either choose to be included in our database to be contacted about future research studies, or you can request that I destroy the information I collect.

You will not be paid for answering questions in this interview since it is only to see whether you qualify to take part in the study, and whether you want to be part of a database of people who are contacted about future studies.

If you have any questions, concerns, or complaints about this interview, contact Dr. Sean Mackey at 650-725-9636. If you want to talk to someone separate from the research team about a concern or complaint or your rights as a possible research subject, please contact the Stanford Institutional Review Board (IRB) to speak to an informed person who is separate from the research team, at 650- 723-5244 or toll-free at 1-866-680-2906. In addition, please call the Stanford IRB at these numbers if you cannot reach the research team.

---

1) Date of Screen ______________________________

---

2) Ok to contact for other studies?
◯ Yes
◯ No

---

3) First name ______________________________

---

4) Last name ______________________________

---

5) Street, City, State, ZIP ______________________________________

---

6) Phone number (cell) ______________________________

---

7) Phone number (home) ______________________________

---

8) Preferred method of contact
☐ Home phone
☐ Cell phone
☐ Email

---

9) Best time to call ______________________________

10) E-mail address ________________________________

11) Date of birth ________________________________

12) Age ________________________________

13) Gender
◯ Female
◯ Male

14) Height (ft) ________________________________

15) Weight (lbs) ________________________________

16) English is your first language.
◯ True
◯ False

17) CRPS
◯ Type I
◯ Type II
◯ RSD/CRPS but don't know the type

18) CRPS Symptoms
☐ Allodynia
☐ Weakness
☐ Hyperalgesia
☐ Tremor
☐ Edema
☐ Dystonia
☐ Decreased Range of Motion
☐ Sweating changes/asymmetry
☐ Trophic changes (hair/nails/skin)

19) Unilateral Upper Extremity
◯ Yes
◯ No

20) Location of pain ________________________________

21) Onset of pain ________________________________

22) NRS Avg Pain Rating ________________________________

23) Cause of pain disorder ________________________________

24) Current Treatments for Pain ________________________________

25) Are you on a stable pain treatment?
◯ Yes
◯ No

26) Will you be trying any new pain treatments in the next 2 months?
◯ Yes
◯ No

27) Additional chronic pain conditions? (Identify each and obtain NRS Avg. for each:)
____

28) History or current psychiatric illness or other condition that would interfere with brain processes (i.e. anxiety, depression, memory and cognition problems)
◯ Yes
◯ No

29) Any ongoing legal action related to pain?
◯ Yes
◯ No

30) Any ongoing disability of worker's compensation claims?
◯ Yes
◯ No

31) If Female: Currently Pregnant or planning to become pregnant?
◯ Yes
◯ No

32) Other notes:
____

33) Ineligible?
____

BL CSS Symptoms (Patient Observed)

Baseline Visit Physical Exam Part I

SYMPTOMS: Answer each as reported by patient over the past 48 hours:

Visit type	◯ Baseline<br>◯ Treatment<br>◯ Follow-up
Today's Date	__________________________________
Diagnosis<br><br>Type 1 = occurs after an illness or injury that didn't directly damage the nerves in your affected limb<br><br>Type 2= follows a distinct nerve injury	◯ CRPS-I<br>◯ CRPS-II<br>◯ CRPS (doesn't remember type)<br>◯ Non-CRPS
Etiology	☐ Crush<br>☐ Surgery<br>☐ Fracture<br>☐ Laceration<br>☐ Soft Tissue Injury<br>☐ Other
Details of CRPS Injury:	____
Date of Injury	__________________________________
Date of symptom onset:	__________________________________
Location	☐ Left<br>☐ Right<br>☐ Upper extremity<br>☐ Lower extremity
Spreading	☐ None (distal only)<br>☐ Proximal limb<br>☐ Mirror image<br>☐ Hemilateral<br>☐ All 4 quadrants
Details of CRPS Location:	____
Are Multiple limbs affected?	◯ Yes ◯ No
If so, how many?	◯ 2 ◯ 3 ◯ 4

Location of affected area #2	☐ Left<br>☐ Right<br>☐ Upper extremity<br>☐ Lower extremity
Comments for Affected Area #2	__________________________________
Location of affected area #3	☐ Left<br>☐ Right<br>☐ Upper extremity<br>☐ Lower extremity
Comments for Affected Area #3	__________________________________
Location of affected area #4	☐ Left<br>☐ Right<br>☐ Upper extremity<br>☐ Lower extremity
Comments for Affected Area #4	__________________________________
Specify most painful area (used for targeting):	__________________________________

CRPS CSS SYMPTOMS: As reported by the patient

Continuing, disproportionate pain:	◯ Yes ◯ No
Comments:	__________________________________

1. Sensory

1a. Allodynia (Non-painful stimuli are painful)<br>For example: clothes, bed sheets, light touch	◯ Yes ◯ No
Comments:	__________________________________
1b: Hyperalgesia (Painful stimuli hurt more than they should)<br>For example: stubbed toe, small cut	◯ Yes ◯ No
Comments:	__________________________________
Hypoesthesia (decreased sensitivity, numbness)	◯ Yes ◯ No
Location of hypoesthesia or comments:	__________________________________

2. Vasomotor

2a. Temperature asymmetry ◯ Yes ◯ No

Specify temperature asymmetry on affected side
◯ Cold
◯ Warm
◯ Labile

Comments: ______________________

2b. Color asymmetry ◯ Yes ◯ No

Specify Color Asymmetry
☐ Red
☐ Blue
☐ Pale
☐ Molted
☐ Scared
☐ Other

Other/Comments: ______________________

3. Sudomotor/Edema

3a. Edema ◯ Yes ◯ No

Comments: ______________________

3b. Sweating Changes ◯ Yes ◯ No

Comments: ______________________

3c. Sweating asymmetry ◯ Yes ◯ No

Comments: ______________________

4. Motor/Trophic

4a. Dystrophic changes (skin, hair, nails) ◯ Yes ◯ No

Specify dystrophic changes
☐ Nails
☐ Hair
☐ Skin

Comments: ______________________

4b. Motor abnormalities (weakness, tremor, dystonia) ◯ Yes ◯ No

Specify motor abnormalities	☐ Weak<br>☐ Tremor<br>☐ Dystonia (involuntary muscle contraction)<br>☐ Myoclonus (twitches or jerks)
Comments:	__________________________
4c. Decreased ROM	◯ Yes ◯ No
Comments:	__________________________

Pain Ratings

Worst CRPS-related Pain in the Last 24 Hours:	◯ 0 = No pain<br>◯ 1<br>◯ 2<br>◯ 3<br>◯ 4<br>◯ 5<br>◯ 6<br>◯ 7<br>◯ 8<br>◯ 9<br>◯ 10 = Worst Imaginable Pain
Average CRPS-related Pain in the Last 24 Hours	◯ 0 = None<br>◯ 1<br>◯ 2<br>◯ 3<br>◯ 4<br>◯ 5<br>◯ 6<br>◯ 7<br>◯ 8<br>◯ 9<br>◯ 10 = Worst Pain Imaginable
Least amount of CRPS-related Pain in the Last 24 Hours:	◯ 0 = None<br>◯ 1<br>◯ 2<br>◯ 3<br>◯ 4<br>◯ 5<br>◯ 6<br>◯ 7<br>◯ 8<br>◯ 9<br>◯ 10 = Worst Pain Imaginable

END OF SURVEY

Sensory Outcome ______________________

Vaso Outcome ______________________

Sudo Outcome ______________________

Motor/Trophic Outcome ______________________

Meets Category Requirements? ______________________

Meets Budapest for Symptoms? ______________________

BL CSS Signs (Examiner)

Part II Physical Exam

SIGNS: As observed by examiner

Visit Type
◯ Baseline
◯ Treatment
◯ Follow-up

1. Sensory
1a. Allodynia

A. Light touch (Camel hair brush) — ◯ Yes ◯ No ◯ Not able to perform test

Pain Rating to Light Touch only
◯ 0 = None
◯ 1
◯ 2
◯ 3
◯ 4
◯ 5
◯ 6
◯ 7
◯ 8
◯ 9
◯ 10 = Worst Pain Imaginable

Comments: ______________________________

Cold (dip tuning fork in water) — ◯ Yes ◯ No ◯ Not able to perform test

Comments: ______________________________

Heat (dip tuning fork in water) — ◯ Yes ◯ No ◯ Not able to perform test

Comments: ______________________________

Vibration
◯ No
◯ Yes
◯ Not able to perform test

B. Hyperalgesia to pinprick — ◯ Yes ◯ No ◯ Not able to perform test

Pain Rating to Pin prick	◯ 0 (No pain)<br>◯ 1<br>◯ 2<br>◯ 3<br>◯ 4<br>◯ 5<br>◯ 6<br>◯ 7<br>◯ 8<br>◯ 9<br>◯ 10 (Worst Imaginable Pain)
Comments:	__________________________
Hypoesthesia (Decreased sensitivity/Numbness) to light touch	◯ Yes ◯ No ◯ Not able to perform test
Comments:	__________________________
C. Pain when move nearest joint	◯ Yes ◯ No
Comments:	__________________________

2. Vasomotor Testing

2a. Color asymmetry	◯ Yes ◯ No
Specify	☐ Red<br>☐ Blue<br>☐ Mottled<br>☐ Scarred
Comments:	__________________________
2b. Temperature asymmetry by palpation	◯ Yes ◯ No ◯ Not able to perform test
Specify temperature asymmetry	◯ Affected Side Cooler<br>◯ Affected Side Warmer
Temperature of Affected Limb (F)	__________________________
Temperature of Unaffected Limb (F)	__________________________
Comments:	__________________________

3. Sudomotor

3a. Sweating asymmetry	◯ Yes ◯ No
Specify sweating asymmetry	◯ Increased on Affected Side<br>◯ Decreased on Affected Side
Comments:	__________________
3b. Edema asymmetry (Swelling)	◯ Yes ◯ No
Comments:	__________________

4. Motor/Trophic Changes

4a. Dystrophic changes	◯ Yes ◯ No
Specify dystrophic changes	☐ Nails<br>☐ Hair<br>☐ Skin (shiny, thin)
Comments:	__________________
4b. Motor Abnormalities	◯ Yes ◯ No
Specify motor abnormalities	☐ Weakness<br>☐ Tremor<br>☐ Dystonia (involuntary muscle contraction)<br>☐ Myoclonus (twitch or jerk)
Comments:	__________________
4c. Decreased ROM (draw circle in air)	◯ Yes ◯ No ◯ Not able to perform test
Comments:	__________________

END OF SURVEY

Sensory Outcome	__________________
Vaso Outcome	__________________
Sudo Outcome	__________________
Motor/Trophic Outcome	__________________

Meets Category Requirements and Budapest for Signs? __________________________________

Dynamometer

Please complete the survey below.

Thank you!

---

132) Trial 1 ______________________

133) Trial 2 ______________________

134) Trial 3 ______________________

IR Thermometer

Please complete the survey below.

Thank you!

Infrared Thermometer Trials
The examiner will use an infrared thermometer to obtain three measurements of the skin surface at the most painful portion of the affected limb as compared to the contralateral portion of the unaffected limb.

135) Infrared thermometer of affected area 1 ________________________

136) Infrared thermometer of affected area 2 ________________________

137) Infrared thermometer of affected area 3 ________________________

138) Infrared thermometer of unaffected area 1 ________________________

139) Infrared thermometer of unaffected area 2 ________________________

140) Infrared thermometer of unaffected area 3 ________________________

Demographics

Please complete the survey below.

Thank you!

Handedness	◯ Right<br>◯ Left
Do you consider yourself to be Hispanic or Latino/Latina?	◯ Yes<br>◯ No
Which of the following groups do you most strongly consider yourself to be a member of?	◯ American Indian / Alaska Native<br>◯ Asian<br>◯ Caucasian<br>◯ Pacific Islander<br>◯ African American<br>◯ Other
Annual income	◯ Less than $10,000<br>◯ $10,000-$19,999<br>◯ $20,000-$29,999<br>◯ $30,000-$39,999<br>◯ $40,000-$49,999<br>◯ $50,000-$59,999<br>◯ $60,000-$69,999<br>◯ $70,000-$79,999<br>◯ $80,000 or more
What is the highest grade or level of school completed or the highest degree obtained?	◯ Grade school/Elementary school (kindergarten through 6th grade)<br>◯ Middle School (through 8th grade)<br>◯ Some High School<br>◯ High School Diploma or GED<br>◯ Some college, no degree<br>◯ Bachelor's degree (e.g., BA, AB, BS, BBA)<br>◯ Associate Degree or vocational certificate<br>◯ Master's degree (e.g., MA, MS, MEng, MEd, MBA)<br>◯ Professional school degree (e.g., MD, DDS, DVM, JD)<br>◯ Doctoral degree (e.g., PhD, EdD)
Are you currently involved in any litigation or worker's compensation claims?	◯ Yes<br>◯ No
Employment status (Choose all that apply)	☐ Part-time employed<br>☐ Full-time employed<br>☐ Student, currently employed<br>☐ Student, not currently employed<br>☐ Full-time homemaker (regardless of marital status)<br>☐ Only temporarily laid off/sick leave or maternity leave<br>☐ Unemployed<br>☐ Looking for work, unemployed<br>☐ Retired<br>☐ Disabled, permanently or temporarily
If unemployed, indicate the main reason for no job:	______________________________

Marital/Partner status	◯ Never married<br>◯ Married<br>◯ Domestic partnership<br>◯ Divorced<br>◯ Separated<br>◯ Widowed

Medical History

Please complete the survey below.

Thank you!

Section 1: Background Information

How did you hear about this study? __________________________________

What is your motivation for participating in this study? __________________________________

Describe your living situation (live alone, significant other, roommates, children, etc): __________________________________

Section 2: Past Medical History

Please list any medical events requiring a visit to an emergency department or urgent care center: __________________________________

Please list any medical conditions requiring hospitalization: __________________________________

Have you had any surgeries/procedures related to pain problem(s)?
- ◯ Yes
- ◯ No

List the name and year of surgery: __________________________________

Have you had other surgeries not related to pain?
- ◯ Yes
- ◯ No

List the name and year of surgery: __________________________________

Section 3: Review of Current Medical Conditions

INSTRUCTIONS: INDICATE CURRENT, UNRESOLVED, OR FREQUENTLY RE-OCCURING CONDITIONS

General Health Conditions	☐ Poor health lately<br>☐ Recent weight change<br>☐ Fever<br>☐ Fatigue<br>☐ No current concerns
Eyes and Vision	☐ Wear glasses or contact lenses<br>☐ Eye disease or injury<br>☐ Blurred or double vision<br>☐ Glaucoma<br>☐ None
Ears, Nose, and Throat	☐ Hearing loss<br>☐ Ringing in the ears<br>☐ Earaches or drainage<br>☐ Sinus problems<br>☐ Bad breath or bad taste<br>☐ Sore throat or voice change<br>☐ None
Heart and Cardiovascular	☐ High blood pressure<br>☐ High cholesterol<br>☐ Heart murmur or irregular beat<br>☐ Chest pains<br>☐ Past heart attack or cardiac failure<br>☐ Swelling of feet, ankles, hands<br>☐ None
Respiratory	☐ Frequent coughing<br>☐ Shortness of breath<br>☐ Asthma or wheezing<br>☐ None
Gastrointestinal	☐ Loss of appetite<br>☐ Change in bowel movements<br>☐ Nausea or vomiting<br>☐ Painful bowel movements<br>☐ Stomach pain<br>☐ None
Genitourinary	☐ Frequent urination<br>☐ Burning or painful urination<br>☐ Kidney stones<br>☐ Painful periods<br>☐ Irregular periods<br>☐ None

Musculoskeletal	☐ Arthritis<br>☐ Joint pain<br>☐ Joint stiffness or swelling<br>☐ Weakness of muscles/joints<br>☐ Muscle pain or cramps<br>☐ Neck/Back pain<br>☐ Cold extremities<br>☐ Difficulty in walking<br>☐ None
Skin	☐ Rash or itching<br>☐ Change in skin color<br>☐ None
Neurological	☐ Frequent or recurrent headaches<br>☐ Light headed or dizzy<br>☐ Convulsions or seizures<br>☐ Numbness or tingling sensations<br>☐ Tremors<br>☐ Paralysis<br>☐ Stroke<br>☐ Head injury<br>☐ None
Psychiatric	☐ Memory loss or confusion<br>☐ Nervousness<br>☐ Sleep problems<br>☐ None
Endocrine	☐ Glandular or hormone problem<br>☐ Thyroid disease<br>☐ Diabetes<br>☐ Excessive thirst or urination<br>☐ Heat or cold intolerance<br>☐ Dry skin<br>☐ Change in hat or glove size<br>☐ None
Hematologic/Lymphatic/Immune	☐ Slow to heal after cuts<br>☐ Easily bruise or bleed<br>☐ Anemia<br>☐ Phlebitis<br>☐ Transfusion<br>☐ Swollen glands<br>☐ HIV/Hepatitis/Tuberculosis<br>☐ Other<br>☐ None
Other Medical Problems?<br>If cancer, list site and status.	______________________________

Medial History Update

Please complete the survey below.

Thank you!

173) Today's Date

____

174) Have you had any major life events since your last visit (such as changes in employment, living situation, major legal/financial concerns, etc)?

____

175) Have you had any major health events since your last visit (such as change in ongoing conditions, new illnesses or injuries, etc)?

____

176) Medications: List all medications you are currently taking (prescriptions, over the counter, herbal, and supplements).

Include Dose/Amount, Frequency, Purpose and Time Last taken for each medication.

Eg. Acetaminophen, 325mg, ~1 per day, backpain, last taken last night

____

177) Other Substances: Please list other substances you have used in the last month (not listed in medication list above).

Include Frequency, Average Amount per Day, Most Amount per Day and Purpose for each substance.

eg. Caffeinated beverages, once a day, 2 cups on average, 4 cups max, habit/alertness.

____

Treatment History

Please complete the survey below.

Thank you!

Treatments:

Please mark below all treatments you have had for your pain problem, then mark how effective it was.

	Had This Treatment in the Past 3 Months	Had This Treatment More Than 3 Months Ago	Never Had This Treatment
Comprehensive Interdisciplinary Program	☐	☐	☐
Physical Therapy	☐	☐	☐
Hot Packs	☐	☐	☐
Ultrasound	☐	☐	☐
Ice	☐	☐	☐
Electrical Stimulation	☐	☐	☐
TENS Unit for Home	☐	☐	☐
Strengthening Exercise	☐	☐	☐
Aerobic Exercise	☐	☐	☐
Traction	☐	☐	☐
Bed Rest	☐	☐	☐
Chiropractic Treatment	☐	☐	☐
Osteopathic Medicine	☐	☐	☐
Biofeedback	☐	☐	☐
Local Injections (Trigger Point)	☐	☐	☐
Back Injections	☐	☐	☐
Acupuncture	☐	☐	☐
Sympathetic Blocks	☐	☐	☐
Ketamine Injection	☐	☐	☐
Massage	☐	☐	☐
Mirror Therapy	☐	☐	☐

Treatment Effect

What was the effect of the treatment?

	No Effect	Helped	Made Worse
Comprehensive Interdisciplinary Program	○	○	○
Physical Therapy	○	○	○
Hot Packs	○	○	○
Ultrasound	○	○	○

Ice	○	○	○
Electrical Stimulation	○	○	○
TENS Unit for Home	○	○	○
Strengthening Exercise	○	○	○
Aerobic Exercise	○	○	○
Traction	○	○	○
Bed Rest	○	○	○
Chiropractic Treatment	○	○	○
Osteopathic Medicine	○	○	○
Biofeedback	○	○	○
Local Injections (Trigger Point)	○	○	○
Back Injections	○	○	○
Acupuncture	○	○	○
Sympathetic Blocks	○	○	○
Ketamine Injection	○	○	○
Massage	○	○	○
Mirror Therapy	○	○	○

Pretreatment Inperson Survey (SETS)

Please complete the survey below.

Thank you!

MOTIVATION AND EXPECTANCY

220) How did you hear about this study? __________________________________

221) What is your motivation for participating in this study? __________________________________

222) Have you ever tried virtual reality before?
- ◯ Yes
- ◯ No

MOTIVATION AND EXPECTANCY

The following questions are about a treatment you will soon receive. We want to know how you think you will respond to that treatment. Please indicate how much you agree with each statement by filling in the appropriate circle. For example, if you strongly disagree with a statement, fill in the circle on the far left.

Your responses to these questions will not affect the treatment you receive in any way. We realize it may be difficult for you to guess how you will respond to a new treatment. If you are unsure about any statement, please give the best guess you can. There are no right or wrong answers.

	Strongly disagree	Moderately disagree	Slightly disagree	Neither agree nor disagree	Slightly agree	Moderately agree	Strongly agree
223) This treatment will be completely effective	○	○	○	○	○	○	○
224) I am worried about my treatment	○	○	○	○	○	○	○
225) My condition will be completely resolved after treatment	○	○	○	○	○	○	○
226) I have fears about this treatment	○	○	○	○	○	○	○
227) I have complete confidence in this treatment	○	○	○	○	○	○	○
228) I am nervous about the negative effects of this treatment	○	○	○	○	○	○	○

Sensory Hypersensitivity Scale (SHS)

Please complete the survey below.

Thank you!

Please complete this questionnaire by selecting the button that best describes your experience.

	Strongly Disagree	Disagree	Neutra/Not Sure	Agree	Strongly Agree
229) I can tolerate hot temperatures better than most people.	○	○	○	○	○
230) I am generally unable to wear clothes made of rough material	○	○	○	○	○
231) I am quite sensitive to pain.	○	○	○	○	○
232) My eyes are sensitive to sunlight.	○	○	○	○	○
233) I often react to odors that others do not initially notice.	○	○	○	○	○
234) I suffer from allergies.	○	○	○	○	○
235) I often feel too hot when others don't seem to be bothered.	○	○	○	○	○
236) I tend to be a picky eater.	○	○	○	○	○
237) I am sensitive to rough textures.	○	○	○	○	○
238) When I read, it must be totally quiet.	○	○	○	○	○
239) I am easily disturbed by high temperatures.	○	○	○	○	○
240) I cannot study or read if there is any conversation or noise around.	○	○	○	○	○
241) I seem to notice smells that other people do not.	○	○	○	○	○
242) I am sensitive to bright light.	○	○	○	○	○
243) I can wear almost any kind of fabric without it bothering me.	○	○	○	○	○
244) I can work even in noisy circumstances.	○	○	○	○	○
245) I can tolerate a large amount of pain.	○	○	○	○	○
246) I am allergy-free.	○	○	○	○	○
247) I am not really bothered by bright lights.	○	○	○	○	○
248) I have a number of allergies.	○	○	○	○	○
249)					

Things that would ordinarily hurt others are not painful to me.	○	○	○	○	○
250) There are many foods that taste bad to me.	○	○	○	○	○
251) I often feel too cold when others don't seem to be bothered.	○	○	○	○	○
252) I can eat almost anything.	○	○	○	○	○
253) I am easily disturbed by low temperatures.	○	○	○	○	○
254) I rarely notice smells.	○	○	○	○	○
255) I can tolerate cold temperatures better than most people.	○	○	○	○	○

Pain Disability Index

Please complete the survey below.

Thank you!

256) Type of Visit?
- ◯ Baseline
- ◯ Treatment
- ◯ Follow-up

257) Today's Date

____

In order to determine how effective your treatment is, we need to know how much pain is interfering in your normal activities. For the 7 areas listed below, please circle the number on the scale which describes the level of disability you have experienced in each area OVER THE PAST WEEK. A score of "0" means no disability at all, and a score of "10" indicates that all of the activities which you would normally do have been totally disrupted or prevented by your pain over the past week. Circle "0" if a category does not apply.

258) Family / Home Responsibilities: This category refers to activities related to the home or family. It includes chores or duties performed around the house (e.g., yard work, house cleaning) and errands or favors for other family members (e.g., driving the children to school).
- ◯ 0 (No Disability)
- ◯ 1
- ◯ 2
- ◯ 3
- ◯ 4
- ◯ 5 (Moderate)
- ◯ 6
- ◯ 7
- ◯ 8
- ◯ 9
- ◯ 10 (Total Disability)

259) Recreation: This category includes hobbies, sports, and other similar leisure time activities.
- ◯ 0 (No Disability)
- ◯ 1
- ◯ 2
- ◯ 3
- ◯ 4
- ◯ 5 (Moderate)
- ◯ 6
- ◯ 7
- ◯ 8
- ◯ 9
- ◯ 10 (Total Disability)

260) Social Activity: This category refers to activities which involve participation with friends and acquaintances other than family members. It includes parties, theater, concerts, dining out, and other social functions.
- ◯ 0 (No Disability)
- ◯ 1
- ◯ 2
- ◯ 3
- ◯ 4
- ◯ 5 (Moderate)
- ◯ 6
- ◯ 7
- ◯ 8
- ◯ 9
- ◯ 10 (Total Disability)

261) Occupation: This category refers to activities that are a part of or directly related to own's job. This includes non-paying jobs as well, such as housewife or volunteer worker.

- ◯ 0 (No Disability)
- ◯ 1
- ◯ 2
- ◯ 3
- ◯ 4
- ◯ 5 (Moderate)
- ◯ 6
- ◯ 7
- ◯ 8
- ◯ 9
- ◯ 10 (Total Disability)

262) Sexual Behavior: This category refers to the frequency and quality of one's sex life.

- ◯ 0 (No Disability)
- ◯ 1
- ◯ 2
- ◯ 3
- ◯ 4
- ◯ 5 (Moderate)
- ◯ 6
- ◯ 7
- ◯ 8
- ◯ 9
- ◯ 10 (Total Disability)

263) Self-Care: This category includes activities which involve personal maintenance and independent daily living (e.g., taking a shower, driving, getting dressed).

- ◯ 0 (No Disability)
- ◯ 1
- ◯ 2
- ◯ 3
- ◯ 4
- ◯ 5 (Moderate)
- ◯ 6
- ◯ 7
- ◯ 8
- ◯ 9
- ◯ 10 (Total Disability)

264) Life-Support Activity: This category refers to basic life-supporting behaviors such as eating and sleeping.

- ◯ 0 (No Disability)
- ◯ 1
- ◯ 2
- ◯ 3
- ◯ 4
- ◯ 5 (Moderate)
- ◯ 6
- ◯ 7
- ◯ 8
- ◯ 9
- ◯ 10 (Total Disability)

PROMIS SF v1.0-Anxiety 8a

Please complete the survey below.

Thank you!

265) In the past 7 days
I felt fearful

- ◯ Never
- ◯ Rarely
- ◯ Sometimes
- ◯ Often
- ◯ Always

266) In the past 7 days
I found it hard to focus on anything other than my anxiety

- ◯ Never
- ◯ Rarely
- ◯ Sometimes
- ◯ Often
- ◯ Always

267) In the past 7 days
My worries overwhelmed me

- ◯ Never
- ◯ Rarely
- ◯ Sometimes
- ◯ Often
- ◯ Always

268) In the past 7 days
I felt uneasy

- ◯ Never
- ◯ Rarely
- ◯ Sometimes
- ◯ Often
- ◯ Always

269) In the past 7 days
I felt nervous

- ◯ Never
- ◯ Rarely
- ◯ Sometimes
- ◯ Often
- ◯ Always

270) In the past 7 days
I felt like I needed help for my anxiety

- ◯ Never
- ◯ Rarely
- ◯ Sometimes
- ◯ Often
- ◯ Always

271) In the past 7 days
I felt anxious

- ◯ Never
- ◯ Rarely
- ◯ Sometimes
- ◯ Often
- ◯ Always

272) In the past 7 days
I felt tense

- ◯ Never
- ◯ Rarely
- ◯ Sometimes
- ◯ Often
- ◯ Always

PROMIS SF v1.0-Depression 8a

Please complete the survey below.

Thank you!

273) In the past 7 days
I felt worthless

- ◯ Never
- ◯ Rarely
- ◯ Sometimes
- ◯ Often
- ◯ Always

274) In the past 7 days
I felt helpless

- ◯ Never
- ◯ Rarely
- ◯ Sometimes
- ◯ Often
- ◯ Always

275) In the past 7 days
I felt depressed

- ◯ Never
- ◯ Rarely
- ◯ Sometimes
- ◯ Often
- ◯ Always

276) In the past 7 days
I felt hopeless

- ◯ Never
- ◯ Rarely
- ◯ Sometimes
- ◯ Often
- ◯ Always

277) In the past 7 days
I felt like a failure

- ◯ Never
- ◯ Rarely
- ◯ Sometimes
- ◯ Often
- ◯ Always

278) In the past 7 days
I felt unhappy

- ◯ Never
- ◯ Rarely
- ◯ Sometimes
- ◯ Often
- ◯ Always

279) In the past 7 days
I felt that I had nothing to look forward to

- ◯ Never
- ◯ Rarely
- ◯ Sometimes
- ◯ Often
- ◯ Always

280) In the past 7 days
I felt that nothing could cheer me up

- ◯ Never
- ◯ Rarely
- ◯ Sometimes
- ◯ Often
- ◯ Always

PROMIS SF v1.0 - Pain Behavior 7a

Please complete the survey below.

Thank you!

---

281) In the past 7 days
When I was in pain I became irritable

- ◯ Had no pain
- ◯ Never
- ◯ Rarely
- ◯ Sometimes
- ◯ Often
- ◯ Always

---

282) In the past 7 days
When I was in pain I grimaced

- ◯ Had no pain
- ◯ Never
- ◯ Rarely
- ◯ Sometimes
- ◯ Often
- ◯ Always

---

283) In the past 7 days
When I was in pain I moved extremely slowly

- ◯ Had no pain
- ◯ Never
- ◯ Rarely
- ◯ Sometimes
- ◯ Often
- ◯ Always

---

284) In the past 7 days
When I was in pain I moved stiffly

- ◯ Had no pain
- ◯ Never
- ◯ Rarely
- ◯ Sometimes
- ◯ Often
- ◯ Always

---

285) In the past 7 days
When I was in pain I called out for someone to help me

- ◯ Had no pain
- ◯ Never
- ◯ Rarely
- ◯ Sometimes
- ◯ Often
- ◯ Always

---

286) In the past 7 days
When I was in pain I isolated myself from others

- ◯ Had no pain
- ◯ Never
- ◯ Rarely
- ◯ Sometimes
- ◯ Often
- ◯ Always

---

287) In the past 7 days
When I was in pain I thrashed

- ◯ Had no pain
- ◯ Never
- ◯ Rarely
- ◯ Sometimes
- ◯ Often
- ◯ Always

PROMIS SF v2.0 - Physical Function 20a

Please complete the survey below.

Thank you!

---

288) Are you able to do chores such as vacuuming or yard work?
- ◯ Without any difficulty
- ◯ With a little difficulty
- ◯ With some difficulty
- ◯ With much difficulty
- ◯ Unable to do

---

289) Are you able to push open a heavy door?
- ◯ Without any difficulty
- ◯ With a little difficulty
- ◯ With some difficulty
- ◯ With much difficulty
- ◯ Unable to do

---

290) Are you able to dress yourself, including tying shoelaces and buttoning your clothes?
- ◯ Without any difficulty
- ◯ With a little difficulty
- ◯ With some difficulty
- ◯ With much difficulty
- ◯ Unable to do

---

291) Are you able to wash your back?
- ◯ Without any difficulty
- ◯ With a little difficulty
- ◯ With some difficulty
- ◯ With much difficulty
- ◯ Unable to do

---

292) Are you able to dry your back with a towel?
- ◯ Without any difficulty
- ◯ With a little difficulty
- ◯ With some difficulty
- ◯ With much difficulty
- ◯ Unable to do

---

293) Are you able to sit on the edge of a bed?
- ◯ Without any difficulty
- ◯ With a little difficulty
- ◯ With some difficulty
- ◯ With much difficulty
- ◯ Unable to do

---

294) Are you able to wash and dry your body?
- ◯ Without any difficulty
- ◯ With a little difficulty
- ◯ With some difficulty
- ◯ With much difficulty
- ◯ Unable to do

---

295) Are you able to get in and out of a car?
- ◯ Without any difficulty
- ◯ With a little difficulty
- ◯ With some difficulty
- ◯ With much difficulty
- ◯ Unable to do

---

296) Are you able to squeeze a new tube of toothpaste?
- ◯ Without any difficulty
- ◯ With a little difficulty
- ◯ With some difficulty
- ◯ With much difficulty
- ◯ Unable to do

297) Are you able to hold a plate full of food?
- ◯ Without any difficulty
- ◯ With a little difficulty
- ◯ With some difficulty
- ◯ With much difficulty
- ◯ Unable to do

298) Are you able to run a short distance, such as to catch a bus?
- ◯ Without any difficulty
- ◯ With a little difficulty
- ◯ With some difficulty
- ◯ With much difficulty
- ◯ Unable to do

299) Are you able to shampoo your hair?
- ◯ Without any difficulty
- ◯ With a little difficulty
- ◯ With some difficulty
- ◯ With much difficulty
- ◯ Unable to do

300) Are you able to sit on and get up from the toilet?
- ◯ Without any difficulty
- ◯ With a little difficulty
- ◯ With some difficulty
- ◯ With much difficulty
- ◯ Unable to do

301) Are you able to transfer from a bed to a chair and back?
- ◯ Without any difficulty
- ◯ With a little difficulty
- ◯ With some difficulty
- ◯ With much difficulty
- ◯ Unable to do

302) Does your health now limit you in doing vigorous activities, such as running, lifting heavy objects, participating in strenuous sports?
- ◯ Not at all
- ◯ Very little
- ◯ Somewhat
- ◯ Quite a lot
- ◯ Cannot do

303) Does your health now limit you in bending, kneeling, or stooping?
- ◯ Not at all
- ◯ Very little
- ◯ Somewhat
- ◯ Quite a lot
- ◯ Cannot do

304) Does your health now limit you in lifting or carrying groceries?
- ◯ Not at all
- ◯ Very little
- ◯ Somewhat
- ◯ Quite a lot
- ◯ Cannot do

305) Does your health now limit you in doing two hours of physical labor?
- ◯ Not at all
- ◯ Very little
- ◯ Somewhat
- ◯ Quite a lot
- ◯ Cannot do

306) Does your health now limit you in walking more than a mile (1.6 km)?
- ◯ Not at all
- ◯ Very little
- ◯ Somewhat
- ◯ Quite a lot
- ◯ Cannot do

307) Does your health now limit you in climbing one flight of stairs?

- ◯ Not at all
- ◯ Very little
- ◯ Somewhat
- ◯ Quite a lot
- ◯ Cannot do

Patient Global Impression Of Change Pgic

Please complete the survey below.

Thank you!

---

308) Since the start of the study, my overall status is:

- ◯ Very Much Improved
- ◯ Much Improved
- ◯ Minimally Improved
- ◯ No Change
- ◯ Minimally Worse
- ◯ Much Worse
- ◯ Very Much Worse

Presence Questions

Please complete the survey below.

Thank you!

Part 1

309) Did you experience any simulator sickness?
- ◯ Not at all
- ◯ Slightly
- ◯ Moderately
- ◯ Strongly
- ◯ Very strongly

310) How natural did your interactions with the environment seem?
- ◯ Not at all
- ◯ Slightly natural
- ◯ Moderately natural
- ◯ Natural
- ◯ Very natural

311) How much did the visual aspects of the environment involve you?
- ◯ Not at all
- ◯ Slightly
- ◯ Moderately
- ◯ Strongly
- ◯ Very strongly

312) How much did your experiences in the virtual environment seem consistent with your real world experiences?
- ◯ Not at all
- ◯ Slightly consistent
- ◯ Moderately consistent
- ◯ Consistent
- ◯ Very consistent

313) How involved were you in the virtual environment experience?
- ◯ Not at all
- ◯ Slightly
- ◯ Moderately
- ◯ Strongly
- ◯ Very strongly

314) How quickly did you adjust to the virtual environment experience?
- ◯ Not at all adjusted
- ◯ Slightly quickly
- ◯ Moderately quickly
- ◯ Quickly
- ◯ Very quikly

315) How compelling was your sense of moving around inside the virtual environment?
- ◯ Not at all
- ◯ Slightly
- ◯ Moderately
- ◯ Strongly
- ◯ Very strongly

316) How proficient in moving and interacting with the virtual environment did you feel at the end of the experience?
- ◯ Not at all proficient
- ◯ Slightly proficient
- ◯ Moderately proficient
- ◯ Proficient
- ◯ Very proficient

317) How much delay did you experience between your actions and expected outcomes?
- ◯ Not at all
- ◯ Slightly
- ◯ Moderately
- ◯ Strongly
- ◯ Very strongly

318) How much did the visual display quality interfere or distract you from performing assigned tasks or required activities?

- ◯ Not at all distracting
- ◯ Slightly distracting
- ◯ Moderately distracting
- ◯ Distracting
- ◯ Very distracting

319) How much did the auditory aspects of the environment involve you?

- ◯ Not at all
- ◯ Slightly
- ◯ Moderately
- ◯ Strongly
- ◯ Very strongly

Part 2

	Not at all	Slightly agree	Moderately agree	Strongly agree	Very Strongly agree
320) If something happened to the avatar, it was happening to me.	◯	◯	◯	◯	◯
321) The avatar's body was my own body.	◯	◯	◯	◯	◯
322) I was in the avatar's body.	◯	◯	◯	◯	◯
323) The avatar was an extension of me.	◯	◯	◯	◯	◯
324) The avatar was me.	◯	◯	◯	◯	◯
325) I was really inside the virtual environment.	◯	◯	◯	◯	◯
326) I felt surrounded by the virtual environment.	◯	◯	◯	◯	◯
327) The virtual lab seemed like the real world.	◯	◯	◯	◯	◯

Daily Pain Survey

Please complete the survey below.

Thank you!

The following questions refer to your PAIN level, ACTIVITY level, MOOD, SLEEP, and PAIN MEDICATION use over the PAST 24 HOURS.

If you have any questions, please feel free to reach me by email or at (650) 4985210.

---

328) Today's Date ______________________________

"Please indicate the intensity of current, best, and worst pain levels over the past 24 hours on a scale of 0 (no pain) to 10 (worst pain imaginable)"

329) How would you rate your average level of pain throughout your body over the past 24 hours?

◯ 0 = None ◯ 1 ◯ 2 ◯ 3 ◯ 4 ◯ 5 ◯ 6 ◯ 7 ◯ 8 ◯ 9 ◯ 10 = Worst Pain Imaginable

330) How would you rate your highest level of pain in your affected CRPS limb over the past 24 hours?

◯ 0 = None ◯ 1 ◯ 2 ◯ 3 ◯ 4 ◯ 5 ◯ 6 ◯ 7 ◯ 8 ◯ 9 ◯ 10 = Worst Pain Imaginable

331) How would you rate your average level of pain in your affected CRPS limb over the past 24 hours?

◯ 0 = None ◯ 1 ◯ 2 ◯ 3 ◯ 4 ◯ 5 ◯ 6 ◯ 7 ◯ 8 ◯ 9 ◯ 10 = Worst Pain Imaginable

332) How would you rate your highest level of pain throughout your body over the past 24 hours?

◯ 0 = None ◯ 1 ◯ 2 ◯ 3 ◯ 4 ◯ 5 ◯ 6 ◯ 7 ◯ 8 ◯ 9 ◯ 10 = Worst Pain Imaginable

333) How would you rate your level of physical activity over the past 24 hours?

◯ Inactive
◯ Mildly Active
◯ Moderately Active
◯ Extremely Active

334) Rate your overall mood for the past 24 hours on a scale of 0 (Very Negative) to 10 (Very Positive).

◯ 0 = Very Negative ◯ 1 ◯ 2 ◯ 3 ◯ 4 ◯ 5 ◯ 6 ◯ 7 ◯ 8 ◯ 9 ◯ 10 = Very Positive

335) How would you rate the quality of your sleep over the past 24 hours?

◯ Very poor
◯ Poor
◯ Fair
◯ Good
◯ Very good

336) Did you take any pain medication over the past 24 hours?

◯ No
◯ Less Than Usual
◯ Standard Amount
◯ More than Usual

337) Please rate your CRPS-related pain right now on a scale of 0 (None) to 10 (Worst Pain Imaginable).

◯ 0 = None ◯ 1 ◯ 2 ◯ 3 ◯ 4 ◯ 5 ◯ 6 ◯ 7 ◯ 8 ◯ 9 ◯ 10 = Worst Pain Imaginable

338) Did you have any major medical events, major life events, or other notes over the past 24 hours that you would like us to know about?

____
(Please describe briefly.)

VR Treatment Report

Please complete the survey below.

Thank you!

Treatment Date	______________________________
Staff Name	☐ Ariana Barreau<br>☐ Mark Gaertner<br>☐ Julia Gionnavi<br>☐ Marrissa Jones
Is lab room booked?	◯ Yes<br>◯ No
Is the VR equipment available and set up?	◯ Yes<br>◯ No
Which controller will the patient be using?	◯ right<br>◯ left
Are the questionnaires prepared on the laptop	◯ Yes<br>◯ No

PRE TREATMENT

Treatment number	◯ 1<br>◯ 2<br>◯ 3<br>◯ 4<br>◯ 5
What is your most painful area at the moment?	☐ CRPS limb ☐ hand<br>☐ wrist ☐ elbow ☐ shoulder<br>☐ other
How would you rate your average level of pain in your affected limb over the past 24 hours? (0 = no pain, 10 = worst imaginable pain)	◯ 0 = No pain ◯ 1 ◯ 2<br>◯ 3 ◯ 4 ◯ 5 ◯ 6<br>◯ 7 ◯ 8 ◯ 9 ◯ 10 = Worst Imaginable Pain
How would you rate your current pain in your affected limb? (0 = no pain, 10 = worst imaginable pain)	◯ 0 (No pain) ◯ 1 ◯ 2<br>◯ 3 ◯ 4 ◯ 5 ◯ 6<br>◯ 7 ◯ 8 ◯ 9 ◯ 10 (Worst Imaginable Pain)

How would you describe your current mood on a scale of 0 (Very Negative) to 10 (Very Positive)?	◯ 0 = very negative ◯ 1 ◯ 2 ◯ 3 ◯ 4 ◯ 5 ◯ 6 ◯ 7 ◯ 8 ◯ 9 ◯ 10 = very positive

POST TREATMENT

Time that patient was in headset (mins)	0 15 30 *(Place a mark on the scale above)*
What is your most painful area at the moment?	☐ CRPS limb ☐ hand ☐ wrist ☐ elbow ☐ shoulder ☐ other
How would you rate your average level of pain in your affected limb over the past 24 hours? (0 = no pain, 10 = worst imaginable pain)	◯ 0 = No pain ◯ 1 ◯ 2 ◯ 3 ◯ 4 ◯ 5 ◯ 6 ◯ 7 ◯ 8 ◯ 9 ◯ 10 = Worst Imaginable Pain
How would you rate your current pain in your affected limb? (0 = no pain, 10 = worst imaginable pain)	◯ 0 (No pain) ◯ 1 ◯ 2 ◯ 3 ◯ 4 ◯ 5 ◯ 6 ◯ 7 ◯ 8 ◯ 9 ◯ 10 (Worst Imaginable Pain)
How would you describe your current mood on a scale of 0 (Very Negative) to 10 (Very Positive)?	◯ 0 = very negative ◯ 1 ◯ 2 ◯ 3 ◯ 4 ◯ 5 ◯ 6 ◯ 7 ◯ 8 ◯ 9 ◯ 10 = very positive
Did you experience any nausea?	◯ Yes ◯ No
Nausea	◯ 0 ◯ 1 ◯ 2 ◯ 3 ◯ 4 ◯ 5 ◯ 6 ◯ 7 ◯ 8 ◯ 9 ◯ 10
Did you experience any disorientation?	◯ Yes ◯ No
Disorientation	◯ 0 ◯ 1 ◯ 2 ◯ 3 ◯ 4 ◯ 5 ◯ 6 ◯ 7 ◯ 8 ◯ 9 ◯ 10
Did you have a headache?	◯ Yes ◯ No
headache (0 no headache, 10 severe headache)	◯ 0 ◯ 1 ◯ 2 ◯ 3 ◯ 4 ◯ 5 ◯ 6 ◯ 7 ◯ 8 ◯ 9 ◯ 10
Other comments	______________________

STANFORD UNIVERSITY Research Consent Form

Protocol Director: Sean Mackey, MD, PhD

Protocol Title: Virtual Reality Mechanisms for treatment of CRPS

Are you participating in any other research studies? _____ Yes _____ No

FOR QUESTIONS ABOUT THE STUDY, CONTACT: Dr. Sean Mackey, MD, PhD at (650) 725-9636, 1070 Arastradero Road, Suite 200, Palo Alto, CA 94304.

DESCRIPTION: You are invited to participate in a research study on using Virtual Reality (VR) Based Mirror Therapy (VRBMT) to modulate pain perception in people with Complex Regional Pain Syndrome (CRPS). We are studying what mechanism of VRBMT may be best able to favorably augment pain perception in patients with CRPS pain.

Visits

You will be asked to come to between 4-6 visits at our lab as follows:

Visit 1: You will be asked to participate in a standardized history and focused physical examination to assess CRPS relevant signs and symptoms. You will also be asked about your past and current CRPS treatments, medical history, and medications. We will also give you questionnaires to assess your levels of pain, emotional response to pain (such as depression or anxiety), and disability, among others. This will be a randomized study and you will have a 33 percent chance of being assigned to any virtual reality mechanisms. All three will require you to wear a virtual reality headset but may vary in the need for arm movement trackers. This visit will last 2-3 hours.

Visits 2-6: VR sessions according to your group and a small set of questionnaires. Each visit will last about 1 hour. The final visit may last longer as it includes a larger set of questionnaires.

Follow up

During the approximately 2 weeks of visits you will be asked to answer a short, 5 minute questionnaire online every day. In the month following your last visit, we will ask you to answer the same questionnaire a few times each week.

RISKS AND BENEFITS: The risks associated with this study are that you may feel uncomfortable answering some of the questions on the questionnaires. You have the right to refuse to answer particular questions. You may also become dizzy and disoriented during the VR session; a research assistant will be with you to make sure you don't fall and hurt yourself as a result.

The benefits which may reasonably be expected to result from this study are that you might feel a reduction in your CRPS pain after the VR sessions. We cannot and do not guarantee or promise that you will receive any benefits from this study.

There is a risk that your pain may increase during your time in the study. If you experience increased pain at any point during the study please inform the research team immediately. You will have the option to remain in the study or withdraw at your discretion.

Your decision whether or not to participate in this study will not affect your employment/medical care.

TIME INVOLVEMENT: Your participation in this experiment will take approximately one and a half months including 4-6 visits lasting 1-3 hours and daily questionnaires lasting about 5 minutes per day.

PAYMENTS: You will receive $20 per hour of visit as payment for your participation, which can amount to $160-$200 total.

Payments may only be made to U.S. citizens, legal resident aliens, and those who have a work eligible visa. You may need to provide your social security number to receive payment.

PARTICIPANT'S RIGHTS: If you have read this form and have decided to participate in this project, please understand your participation is voluntary and you have the right to withdraw your consent or discontinue participation at any time without penalty or loss of benefits to which you are otherwise entitled.
The results of this research study may be presented at scientific or professional meetings or published in scientific journals. However, your identity will not be disclosed.
You have the right to refuse to answer particular questions.

Authorization To Use Your Health Information For Research Purposes

Because information about you and your health is personal and private, it generally cannot be used in this research study without your written authorization. If you sign this form, it will provide that authorization. The form is intended to inform you about how your health information will be used or disclosed in the study. Your information will only be used in accordance with this authorization form and the informed consent form and as required or allowed by law. Please read it carefully before signing it.

What is the purpose of this research study and how will my health information be utilized in the study?

This study is being done to assess the use of Virtual Reality (VR) Based Mirror Therapy (VRBMT) in people with Complex Regional Pain Syndrome (CRPS). We are studying what mechanism of VRBMT may be best to favorably augment CRPS pain. Your health information will be used to evaluate these different VRBMT mechanisms. Your health information may be presented at scientific meetings and may be used in publication, but your identity will not be disclosed.

Do I have to sign this authorization form?

You do not have to sign this authorization form. But if you do not, you will not be able to participate in this research study including receiving any research-related treatment. Signing the form is not a condition for receiving any medical care outside the study.

If I sign, can I revoke it or withdraw from the research later?

If you decide to participate, you are free to withdraw your authorization regarding the use and disclosure of your health information (and to discontinue any other participation in the study) at any time. After any revocation, your health information will no longer be used or disclosed in the study, except to the extent that the law allows us to continue using your information (e.g., necessary to maintain integrity of research). If you wish to revoke your authorization for the research use or disclosure of your health information in this study, you must write to Dr. Sean Mackey, MD, PhD at 1070 Arastradero Road, Suite 200, Palo Alto, CA 94304.

What Personal Information Will Be Obtained, Used or Disclosed?

Your health information related to this study, may be used or disclosed in connection with this research study, including, but not limited to, your name, contact info, date of birth, basic demographic information, your medical history, medication history, the information you provide on questionnaires, and the results of your physical exam.

Who May Use or Disclose the Information?

The following parties are authorized to use and/or disclose your health information in connection with this research study:

- The Protocol Director Sean Mackey, MD, PhD
- The Stanford University Administrative Panel on Human Subjects in Medical Research and any other unit of Stanford University as necessary
- Research Staff

Who May Receive or Use the Information?

The parties listed in the preceding paragraph may disclose your health information to the following persons and organizations for their use in connection with this research study:

- The Office for Human Research Protections in the U.S. Department of Health and Human Services

Your information may be re-disclosed by the recipients described above, if they are not required by law to protect the privacy of the information.

When will my authorization expire?

Your authorization for the use and/or disclosure of your health information will end on December 31st, 2050, or when the research project ends, whichever is earlier.

Will access to my medical record be limited during the study?

To maintain the integrity of this research study, you may not have access to any health information developed as part of this study until it is completed. At that point, you would have access to such health information if it was used to make a medical or billing decision about you (e.g., if included in your official medical record).

________________________________ ________________

Signature of Adult Participant Date

____

Name of Adult Participant

WITHDRAWAL FROM STUDY

The Protocol Director may also withdraw you from the study without your consent for one or more of the following reasons:

- Failure to follow the instructions of the Protocol Director and study staff.
- The Protocol Director decides that continuing your participation could be harmful to you.
- Pregnancy
- You need treatment not allowed in the study.
- The study is cancelled.
- Other administrative reasons.
- Unanticipated circumstances.

CONTACT INFORMATION:

If you have any questions, concerns or complaints about this research study, its procedures, risks and benefits, or alternative courses of treatment, you should ask the Protocol Director, Sean Mackey, MD, PhD at (650) 725-9636. You should also contact him at any time if you feel you have been hurt by being a part of this study.

Independent Contact: If you are not satisfied with how this study is being conducted, or if you have any concerns, complaints, or general questions about the research or your rights as a participant, please contact the Stanford Institutional Review Board (IRB) to speak to someone independent of the research team at (650)-723-5244 or toll free at 1-866-680-2906. You can also write to the Stanford IRB, Stanford University, 3000 El Camino Real, Five Palo Alto Square, 4th Floor, Palo Alto, CA 94306.

Appointment Contact: If you need to change your appointment, please contact Ariana Barreau at +1 6504600189.

The extra copy of this signed and dated consent form is for you to keep.

__________________________________ _________________

Signature of Adult Participant Date

____

Name of Adult Participant

Daily Pain Survey

Please complete the survey below.

Thank you!

The following questions refer to your PAIN level, ACTIVITY level, MOOD, SLEEP, and PAIN MEDICATION use over the PAST 24 HOURS.

If you have any questions, please feel free to reach me by email or at (650) 4985210.

---

1) Today's Date

____

"Please indicate the intensity of current, best, and worst pain levels over the past 24 hours on a scale of 0 (no pain) to 10 (worst pain imaginable)"

2) How would you rate your average level of pain throughout your body over the past 24 hours?

○ 0 = None ○ 1 ○ 2 ○ 3 ○ 4 ○ 5 ○ 6 ○ 7 ○ 8 ○ 9 ○ 10 = Worst Pain Imaginable

3) How would you rate your highest level of pain in your affected CRPS limb over the past 24 hours?

○ 0 = None ○ 1 ○ 2 ○ 3 ○ 4 ○ 5 ○ 6 ○ 7 ○ 8 ○ 9 ○ 10 = Worst Pain Imaginable

4) How would you rate your average level of pain in your affected CRPS limb over the past 24 hours?

○ 0 = None ○ 1 ○ 2 ○ 3 ○ 4 ○ 5 ○ 6 ○ 7 ○ 8 ○ 9 ○ 10 = Worst Pain Imaginable

5) How would you rate your highest level of pain throughout your body over the past 24 hours?

○ 0 = None ○ 1 ○ 2 ○ 3 ○ 4 ○ 5 ○ 6 ○ 7 ○ 8 ○ 9 ○ 10 = Worst Pain Imaginable

6) How would you rate your level of physical activity over the past 24 hours?

○ Inactive
○ Mildly Active
○ Moderately Active
○ Extremely Active

7) Rate your overall mood for the past 24 hours on a scale of 0 (Very Negative) to 10 (Very Positive).

○ 0 = Very Negative ○ 1 ○ 2 ○ 3 ○ 4 ○ 5 ○ 6 ○ 7 ○ 8 ○ 9 ○ 10 = Very Positive

8) How would you rate the quality of your sleep over the past 24 hours?

○ Very poor
○ Poor
○ Fair
○ Good
○ Very good

9) Did you take any pain medication over the past 24 hours?

○ No
○ Less Than Usual
○ Standard Amount
○ More than Usual

10) Please rate your CRPS-related pain right now on a scale of 0 (None) to 10 (Worst Pain Imaginable).

○ 0 = None ○ 1 ○ 2 ○ 3 ○ 4 ○ 5 ○ 6 ○ 7 ○ 8 ○ 9 ○ 10 = Worst Pain Imaginable

11) Did you have any major medical events, major life events, or other notes over the past 24 hours that you would like us to know about?

____
(Please describe briefly.)

PROMIS SF v1.0 - Pain Behavior 7a

Please complete the survey below.

Thank you!

1) In the past 7 days
When I was in pain I became irritable

- ◯ Had no pain
- ◯ Never
- ◯ Rarely
- ◯ Sometimes
- ◯ Often
- ◯ Always

2) In the past 7 days
When I was in pain I grimaced

- ◯ Had no pain
- ◯ Never
- ◯ Rarely
- ◯ Sometimes
- ◯ Often
- ◯ Always

3) In the past 7 days
When I was in pain I moved extremely slowly

- ◯ Had no pain
- ◯ Never
- ◯ Rarely
- ◯ Sometimes
- ◯ Often
- ◯ Always

4) In the past 7 days
When I was in pain I moved stiffly

- ◯ Had no pain
- ◯ Never
- ◯ Rarely
- ◯ Sometimes
- ◯ Often
- ◯ Always

5) In the past 7 days
When I was in pain I called out for someone to help me

- ◯ Had no pain
- ◯ Never
- ◯ Rarely
- ◯ Sometimes
- ◯ Often
- ◯ Always

6) In the past 7 days
When I was in pain I isolated myself from others

- ◯ Had no pain
- ◯ Never
- ◯ Rarely
- ◯ Sometimes
- ◯ Often
- ◯ Always

7) In the past 7 days
When I was in pain I thrashed

- ◯ Had no pain
- ◯ Never
- ◯ Rarely
- ◯ Sometimes
- ◯ Often
- ◯ Always

Acknowledgment: PROMIS Health Organization and Assessment Center℠ View full acknowledgment

PROMIS SF v2.0 - Physical Function 20a

Please complete the survey below.

Thank you!

1) Are you able to do chores such as vacuuming or yard work?
- ◯ Without any difficulty
- ◯ With a little difficulty
- ◯ With some difficulty
- ◯ With much difficulty
- ◯ Unable to do

2) Are you able to push open a heavy door?
- ◯ Without any difficulty
- ◯ With a little difficulty
- ◯ With some difficulty
- ◯ With much difficulty
- ◯ Unable to do

3) Are you able to dress yourself, including tying shoelaces and buttoning your clothes?
- ◯ Without any difficulty
- ◯ With a little difficulty
- ◯ With some difficulty
- ◯ With much difficulty
- ◯ Unable to do

4) Are you able to wash your back?
- ◯ Without any difficulty
- ◯ With a little difficulty
- ◯ With some difficulty
- ◯ With much difficulty
- ◯ Unable to do

5) Are you able to dry your back with a towel?
- ◯ Without any difficulty
- ◯ With a little difficulty
- ◯ With some difficulty
- ◯ With much difficulty
- ◯ Unable to do

6) Are you able to sit on the edge of a bed?
- ◯ Without any difficulty
- ◯ With a little difficulty
- ◯ With some difficulty
- ◯ With much difficulty
- ◯ Unable to do

7) Are you able to wash and dry your body?
- ◯ Without any difficulty
- ◯ With a little difficulty
- ◯ With some difficulty
- ◯ With much difficulty
- ◯ Unable to do

8) Are you able to get in and out of a car?
- ◯ Without any difficulty
- ◯ With a little difficulty
- ◯ With some difficulty
- ◯ With much difficulty
- ◯ Unable to do

9) Are you able to squeeze a new tube of toothpaste?
- ◯ Without any difficulty
- ◯ With a little difficulty
- ◯ With some difficulty
- ◯ With much difficulty
- ◯ Unable to do

10) Are you able to hold a plate full of food?

◯ Without any difficulty
◯ With a little difficulty
◯ With some difficulty
◯ With much difficulty
◯ Unable to do

11) Are you able to run a short distance, such as to catch a bus?

◯ Without any difficulty
◯ With a little difficulty
◯ With some difficulty
◯ With much difficulty
◯ Unable to do

12) Are you able to shampoo your hair?

◯ Without any difficulty
◯ With a little difficulty
◯ With some difficulty
◯ With much difficulty
◯ Unable to do

13) Are you able to sit on and get up from the toilet?

◯ Without any difficulty
◯ With a little difficulty
◯ With some difficulty
◯ With much difficulty
◯ Unable to do

14) Are you able to transfer from a bed to a chair and back?

◯ Without any difficulty
◯ With a little difficulty
◯ With some difficulty
◯ With much difficulty
◯ Unable to do

15) Does your health now limit you in doing vigorous activities, such as running, lifting heavy objects, participating in strenuous sports?

◯ Not at all
◯ Very little
◯ Somewhat
◯ Quite a lot
◯ Cannot do

16) Does your health now limit you in bending, kneeling, or stooping?

◯ Not at all
◯ Very little
◯ Somewhat
◯ Quite a lot
◯ Cannot do

17) Does your health now limit you in lifting or carrying groceries?

◯ Not at all
◯ Very little
◯ Somewhat
◯ Quite a lot
◯ Cannot do

18) Does your health now limit you in doing two hours of physical labor?

◯ Not at all
◯ Very little
◯ Somewhat
◯ Quite a lot
◯ Cannot do

19) Does your health now limit you in walking more than a mile (1.6 km)?

◯ Not at all
◯ Very little
◯ Somewhat
◯ Quite a lot
◯ Cannot do

20) Does your health now limit you in climbing one flight of stairs?

- ◯ Not at all
- ◯ Very little
- ◯ Somewhat
- ◯ Quite a lot
- ◯ Cannot do

Acknowledgment: PROMIS Health Organization and Assessment Center℠ View full acknowledgment

PROMIS SF v1.0-Anxiety 8a

Please complete the survey below.

Thank you!

1) In the past 7 days
I felt fearful

◯ Never
◯ Rarely
◯ Sometimes
◯ Often
◯ Always

2) In the past 7 days
I found it hard to focus on anything other than my anxiety

◯ Never
◯ Rarely
◯ Sometimes
◯ Often
◯ Always

3) In the past 7 days
My worries overwhelmed me

◯ Never
◯ Rarely
◯ Sometimes
◯ Often
◯ Always

4) In the past 7 days
I felt uneasy

◯ Never
◯ Rarely
◯ Sometimes
◯ Often
◯ Always

5) In the past 7 days
I felt nervous

◯ Never
◯ Rarely
◯ Sometimes
◯ Often
◯ Always

6) In the past 7 days
I felt like I needed help for my anxiety

◯ Never
◯ Rarely
◯ Sometimes
◯ Often
◯ Always

7) In the past 7 days
I felt anxious

◯ Never
◯ Rarely
◯ Sometimes
◯ Often
◯ Always

8) In the past 7 days
I felt tense

◯ Never
◯ Rarely
◯ Sometimes
◯ Often
◯ Always

Acknowledgment: PROMIS Health Organization and Assessment Center℠ View full acknowledgment

Presence Questions

Please complete the survey below.

Thank you!

Part 1

1) Did you experience any simulator sickness?
   - ◯ Not at all
   - ◯ Slightly
   - ◯ Moderately
   - ◯ Strongly
   - ◯ Very strongly

2) How natural did your interactions with the environment seem?
   - ◯ Not at all
   - ◯ Slightly natural
   - ◯ Moderately natural
   - ◯ Natural
   - ◯ Very natural

3) How much did the visual aspects of the environment involve you?
   - ◯ Not at all
   - ◯ Slightly
   - ◯ Moderately
   - ◯ Strongly
   - ◯ Very strongly

4) How much did your experiences in the virtual environment seem consistent with your real world experiences?
   - ◯ Not at all
   - ◯ Slightly consistent
   - ◯ Moderately consistent
   - ◯ Consistent
   - ◯ Very consistent

5) How involved were you in the virtual environment experience?
   - ◯ Not at all
   - ◯ Slightly
   - ◯ Moderately
   - ◯ Strongly
   - ◯ Very strongly

6) How quickly did you adjust to the virtual environment experience?
   - ◯ Not at all adjusted
   - ◯ Slightly quickly
   - ◯ Moderately quickly
   - ◯ Quickly
   - ◯ Very quikly

7) How compelling was your sense of moving around inside the virtual environment?
   - ◯ Not at all
   - ◯ Slightly
   - ◯ Moderately
   - ◯ Strongly
   - ◯ Very strongly

8) How proficient in moving and interacting with the virtual environment did you feel at the end of the experience?
   - ◯ Not at all proficient
   - ◯ Slightly proficient
   - ◯ Moderately proficient
   - ◯ Proficient
   - ◯ Very proficient

9) How much delay did you experience between your actions and expected outcomes?

- ◯ Not at all
- ◯ Slightly
- ◯ Moderately
- ◯ Strongly
- ◯ Very strongly

10) How much did the visual display quality interfere or distract you from performing assigned tasks or required activities?

- ◯ Not at all distracting
- ◯ Slightly distracting
- ◯ Moderately distracting
- ◯ Distracting
- ◯ Very distracting

11) How much did the auditory aspects of the environment involve you?

- ◯ Not at all
- ◯ Slightly
- ◯ Moderately
- ◯ Strongly
- ◯ Very strongly

Part 2

		Not at all	Slightly agree	Moderately agree	Strongly agree	Very Strongly agree
12)	If something happened to the avatar, it was happening to me.	○	○	○	○	○
13)	The avatar's body was my own body.	○	○	○	○	○
14)	I was in the avatar's body.	○	○	○	○	○
15)	The avatar was an extension of me.	○	○	○	○	○
16)	The avatar was me.	○	○	○	○	○
17)	I was really inside the virtual environment.	○	○	○	○	○
18)	I felt surrounded by the virtual environment.	○	○	○	○	○
19)	The virtual lab seemed like the real world.	○	○	○	○	○

Acknowledgment: PROMIS Health Organization and Assessment Center℠ View full acknowledgment
