# Supplementary figures and images for: Assessing the Feasibility of an Open-Source Virtual Reality Mirror Visual Feedback Module for Complex Regional Pain Syndrome: Pilot Usability Study
Source: J Med Internet Res. 2021 May 26;23(5):e16536. doi: 10.2196/16536 (PMC8190641; doi:10.2196/16536)

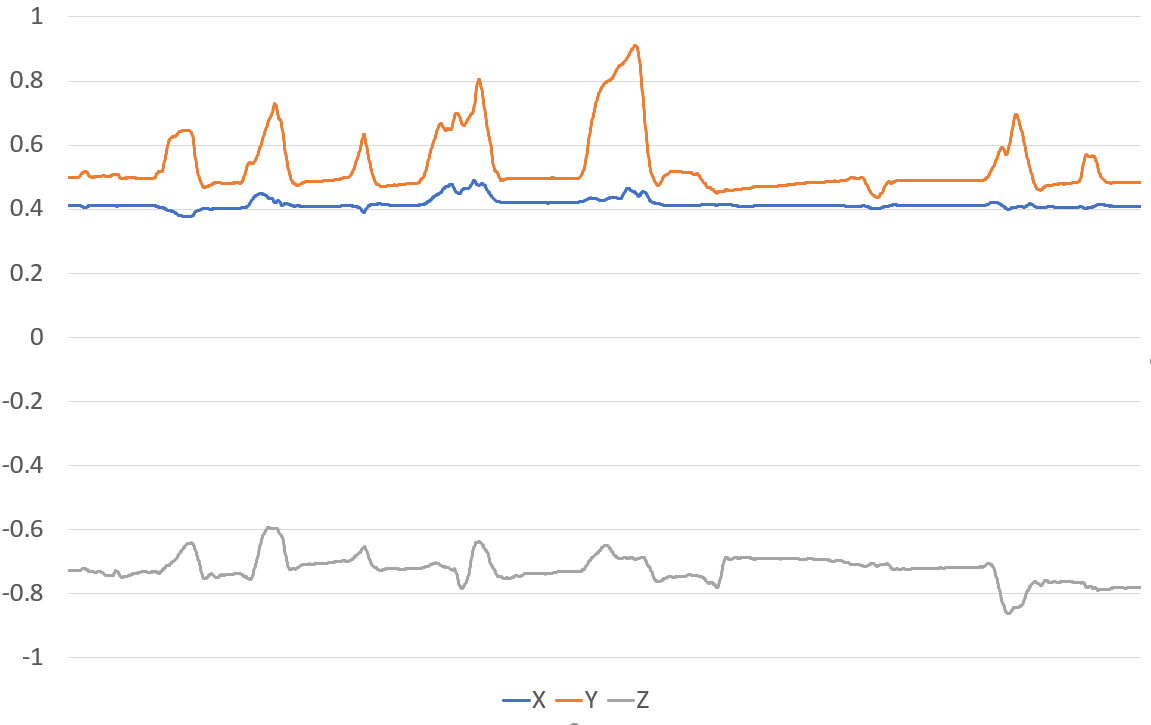

Supplement: Multimedia Appendix 4 [file jmir_v23i5e16536_app4.png]
